# Bacterial isolates and their antibiotic susceptibility patterns among patients with pus and/or wound discharge at Gondar university hospital

**DOI:** 10.1186/1756-0500-7-619

**Published:** 2014-09-09

**Authors:** Dagnachew Muluye, Yitayih Wondimeneh, Getachew Ferede, Tesfaye Nega, Kasaw Adane, Belete Biadgo, Habtie Tesfa, Feleke Moges

**Affiliations:** School of Biomedical and Laboratory Sciences, College of Medicine and Health Sciences, University of Gondar, P.O. Box 196, Gondar, Ethiopia; Unit of Bacteriology, Gondar University Hospital, P.O. Box 196, Gondar, Ethiopia

**Keywords:** Pus, Wound discharge, Gondar University Hospital

## Abstract

**Background:**

In spite of advances in control of infections, wound infections have not completely controlled due to many reasons. The widespread uses of antibiotics, together with the length of time over which they have been available have led to major problems of resistant organisms contributing to morbidity and mortality. This study was aimed to assess bacterial isolates and their drug susceptibility patterns from patients with pus and/or wound discharge.

**Methods:**

A retrospective study was conducted at Gondar University Hospital from all individuals who provide pus and/or wound discharge sample from September, 2009 to August, 2012. Socio-demographic and laboratory results were collected from the University Hospital Microbiology Laboratory unit registration books by using a standard data collection format. Data were analyzed using SPSS version 20 software. P-value ≤ 0.05 was considered statistically significant.

**Result:**

A total of 628 study subjects were included in the study with bacterial isolation rate of 441 (70.2%). Of all, 344 (54.8%) were males. Two hundred eighty two (63.9%) of the isolates were gram positive and 159 (36.1%) were gram negative. About 331/ 441 (75.0%) of the total isolates were *Staphylococcus aureus* (32.9%), Coagulase Negative staphylococci (14.7%), *Streptococcus* spp. (11.6%), *Escherichia coli* (9.5%), *Klebsiella* spp. (6.3%). The result showed that 66.2% of the isolates were resistant to tetracycline, followed 59.8% for ampicillin, 59.1% for cotrimoxazole, 51.7% for penicillin; least resistant being 6.3% for gentamycin.

**Conclusion:**

High prevalence of bacterial isolates were found; *S. aureus* being the dominant. Most of the isolates were resistant to many of the antibiotics tested where all isolates of *Pseudomonas* spp*.* being resistant to two or more antibiotics. Antibiotic susceptibility test is necessary for effective control of wound infections.

## Background

Infectious diseases still remain an important cause of morbidity and mortality among humans, especially in developing countries. Various species of bacteria live on human skin, gastrointestinal tract, in the nasopharynx and other parts of the body with less potential for causing disease because of first line defense within the body. Skin abrasion due to surgical procedure, trauma, burns, diseases, nutrition and other factors affect this first line defense and leads to microbial contamination results infections [1]. As wound infections are largely hospital acquired and the infecting pathogens not only differ from country to country, but also vary from one hospital to another within the same country [2].

The problem of hospital acquired infection remains a serious health hazard worldwide. As described by World Health Organization (WHO) [3], it is one of the major sources of infectious diseases which results for the huge economic impact with significant rate of morbidity and mortality. Despite advances in control of infections, wound infections have not completely prevented due to the problem of drug resistance [4]. The widespread uses of antibiotics, together with the length of time over which they have been available, have led to major problems of resistant organisms contributing to morbidity and mortality [5].

Knowledge of the causative agents of wound infection has proven to be helpful in the selection of appropriate antimicrobial therapy and on infection control measures taken in health institutions [6]. Therefore, the present study aimed to evaluate the important causative agents of bacteria and their antibiotic susceptibility patterns which may be imperative as it guides the selection of an effective regimen for the treatment.

## Methods

A retrospective study was conducted at Gondar University Hospital from September, 2009 to August, 2012. Gondar University Hospital is a teaching hospital that provides health service to over five million inhabitants in Northwest Ethiopia. All individuals who provide pus and/or wound discharge sample at Gondar University Hospital during the study period were the participants. Socio-demographic and laboratory results were collected from the University Hospital Microbiology Laboratory unit registration books by using a standard data collection format after checking the completeness of the data. Wound swabs and discharges were aseptically collected using sterile swab in a test tube and inoculated on to chocolate agar (incubated in CO_2_ enriched environment), blood agar and MacConkey agar. Plates were incubated at 37°C of 24 hours. Once pure colonies identified series of biochemical tests were performed for the isolation of the species following standard procedures [7].

### Antimicrobial susceptibility tests

Antimicrobial susceptibility testing was performed using Muller-Hinton agar (Mumbai, India) plates as recommended by the Clinical and Laboratory Standards Institute (CLSI) [8]. The isolates were tested against chloramphenicol (30 μg), ampicillin (10 μg), ciprofloxacin (5 μg), gentamicin (10 μg), cotrimoxazole (5 μg), tetracycline (30 μg), nalldixic acid (30 μg), Ceftriaxone (30 μg), Norfloxacin (30 μg). For gram-positive isolates, erythromycin (15 μg), methicillin (5 μg), vancomycin, (30 μg), streptomycin (10 μg) were included. Plates were incubated at 35-37°C for 24 hours. The zones of inhibition were measured and compared with the guidelines [8].

### Data analysis

Data were cleaned manually and entered and analyzed by using SPSS version 20 software. Odds ratio and Chi-square test were employed. P-value ≤ 0.05 was considered statistically significant.

### Ethical considerations

Ethical clearance was obtained from the Institutional Review Board of University of Gondar. A supportive letter was also obtained from College of Medicine and Health Sciences and the University Hospital clinical director before collecting the data.

## Results

A total of 628 study subjects were included in the study. Among these, 284 (45.2%) were females and 344 (54.8%) were males with the age range of 1 month to 81 years. Among 628 study subjects, bacterial pathogens were isolated from 441 patients with the isolation rate of 70.2% [Table 1]. Culture positivity rate from 2009-2012 in the bacteriology laboratory of the present study demonstrate the maximum isolation rate in 2009 which was 73.9% and a slightly lower in 2012 which was 69.3% [Table 1]. Two hundred eighty two (63.9%) of the isolates were gram positive and 159 (36.1%) were gram negative. About 331/ 441 (75. 0%) of the total isolates were *Staphylococcus aureus*, Coagulase Negative Staphylococci (CNS), *Streptococcus* spp, *Escherichia coli* and *Klebsiella* spp. Among these *S. aureus* accounts 32.9% followed by CNS (14.7%), *Streptococcus* spp. (11.6%), *E. coli* (9.5%), *Klebsiella* spp. (6.3%), *streptococcus pyogenes* (4.8%), *Pseudomonas* spp*.* (1.8%).

**Table 1:** **Total pus and/or wound discharge and cultured positivity rate at Gondar University Hospital, 2009-2012**

Year assessed		Type of sample (%)		Culture status (%)		
		Wound swab	Pus/discharge	Positive	Negative	Total
**Year**	**2009**	114 (72.6)	43 (27.4)	116 (73.9)	41 (26.1)	157 (100)
	**2010**	148 (73.3)	54 (26.7)	144 (71.3)	58 (28.7)	202 (100)
	**2011**	96 (61.9)	59 (38.1)	102 (65.8)	53 (34.2)	155 (100)
	**2012**	62 (54.4)	52 (45.6)	79 (69.3)	35 (30.7)	114 (100)
	**Total**	420 (66.9)	208 (33.1)	441 (70.2)	187 (29.8)	628 (100)

Most of the isolates were resistant to many of the antibiotics tested. The result showed that 66.2% of the isolates were resistant to tetracycline, followed 59.8% for ampicillin, 59.1% for cotrimoxazole, 51.7% for penicillin; least resistant being 6.3% for gentamycin. Among isolates tested, 18.3% of the isolates are becoming resistant to ceftriaxone and 33.3% for vancomycin [Table 2]. Only twenty isolates of *S. aureus* were tested for Methicillin susceptibility, of which, 4 (20%) were resistant and 16 (80%) were sensitive.

**Table 2:** **Antibiotic susceptibility patterns of isolates from pus and/or wound discharge for commonly used antibiotics at Gondar University Hospital, 2009-2012**

Culture results	Antibiotic susceptibility patterns (%)												
	AMP	CRO	CAF	CIP	E	CN	MET	NOR	PG	SXT	TTC	VAN	NA
**Susceptible**	151 (40.2)	312 (81.7)	240 (83.3)	305 (76.3)	165 (65.2)	192 (93.7)	30 (69.8)	194 (74.6)	116 (48.3)	140 (40.9)	101 (33.8)	20 (66.7)	31 (53.4)
**Resistance**	225 (59.8)	70 (18.3)	48 (16.7)	95 (23.7)	88 (34.8)	13 (6.3)	13 (30.2)	66 (25.4)	124 (51.7)	202 (59.1)	198 (66.2)	10 (33.3)	27 (46.6)
**Total**	376 (100)	382 (100)	288 (100)	400 (100)	253 (100)	205 (100)	43 (100)	260 (100)	240 (100)	342 (100)	299 (100)	30 (100)	58 (100)

AMP = Ampicillin, CRO = Ceftriaxone, CAF = Chloramphinicol, CIP = Ciprofloxacin, E = Erytromycin, CN = Gentamycin; MET = Methicillin, NOR **=** Norfloxacin, PG = Penicillin, SXT = Cotrimoxazole; TTC = Tetracycline; VAN = Vancomycin, NA = Nalidixic acid.

Among resistant isolates to more than one antibiotic, the most common pathogen was *Pseudomonas* spp, (100%); followed by *S. pyogenes* (85.8%); *Enterobacter* spp. (84.2%); and *E. coli* (83.4%). Fifty (11.3%) of the isolates were sensitive to all drugs tested and 68 (15.4%) were resistance to only one drug. However, among the total isolates, 323 (73.3%) of them were resistant for two or more drugs tested and 91 (20.6%) were resistant to more than 5 antibiotics tested. All isolates (100%) of *Pseudomonas* spp. were resistant to 2 or more antibiotics followed by *S. pyogenes 85.8%, Enterobacter* spp. 84.2%, *E.coli* 83.4%, *Streptococcus* spp., 82.3%, *S. aureus* 67.6% [Table 3].

**Table 3:** **Multiple drug resistance patterns of isolates from pus and/or wound discharge at Gondar University Hospital, 2009-2012**

Bacterial isolates	Multiple drug resistance patterns of isolates n (%)							
	Ro	R1	R2	R3	R4	≥ R5	Total	MDR*
*S. aureus*	19 (13.1)	28 (19.3)	41 (28.2)	33 (22.8)	11 (7.6)	13 (9)	145 (100)	98 (67.6)
*E. coli*	4 (9.5)	3 (7.1)	10 (23.8)	7 (16.7)	7 (16.7)	11 (26.2)	42 (100)	35 (83.4)
CNS	12 (18.5)	9 (13.8)	13 (20.0)	7 (10.8)	6 (9.2)	18 (27.7)	65 (100)	41 (67.7)
*Streptococcus* spp.	3 (5.9)	6 (11.8)	9 (17.6)	13 (25.5)	10 (19.6)	10 (19.6)	51 (100)	42 (82.3)
*Proteus* spp.	4 (20)	3 (15)	3 (15)	7 (35)	0	3 (15)	20 (100)	13 (65)
*Klebsiella* spp.	3 (10.7)	4 (14.3)	6 (21.4)	4 (14.3)	1 (3.6)	10 (35.7)	28 (100)	21 (75)
*Enterobacter* spp.	0	3 (15.8)	0	4 (21.1)	2 (10.5)	10 (52.6)	19 (100)	16 (84.2)
*Pseudomonas* spp.	0	0	1 (7.7)	3 (23.1)	4 (30.8)	5 (38.4)	13 (100)	13 (100)
*Shigella* spp.	0	2 (25.0)	2 (25.0)	0	1 (12.5)	3 (37.5)	8 (100)	5 (62.5)
*S. pyogenes*	1 (4.7)	2 (9.5)	4 (19.1)	10 (47.6)	1 (4.8)	3 (14.3)	21 (100)	18 (85.8)
Non-lactose fermentor G-ve rods	4 (13.8)	8 (27.6)	6 (20.7)	5 (17.2)	1 (3.4)	5 (17.3)	29 (100)	17 (58.6)
**Total**	**50 (11.3)**	**68 (15.4)**	**95 (21.6)**	**93 (21.1)**	**44 (10.0)**	**91(20.6)**	**441 (100)**	**323 (73.3)**

*MDR = Resistance to two or more antibiotics.

The majority of the isolates were in the age range of 21 to 30 years which was 113 (25.6%) followed by in the age range of 1-10 years which was 95 (21.5%); age range of 11-20 which was 70 (15.9%) [Table 4].

**Table 4:** **Bacterial isolates from pus and/or wound discharge based on age and sex category from patients at Gondar University Hospital, 2009-2012**

Isolates	Sex		Age range in year (s)								
	Male	Female	< 1	1-10	11-20	21-30	31-40	41-50	51-60	>60	Total
*S. aureus*	74	71	18	32	34	36	9	13	2	1	145
CNS	23	42	5	15	8	13	10	7	3	4	65
*Streptococcus* spp.	24	27	4	15	7	12	3	6	1	3	51
*E. coli*	17	25	4	5	5	14	2	7	4	1	42
NLFGNR	11	18	5	7	2	7	4	3	1	0	29
*Klebsiella* spp.	9	19	3	9	5	4	3	1	1	2	28
*S. pyogenes*	11	10	1	2	3	11	0	4	0	0	21
*Proteus* spp	9	11	0	3	3	6	3	1	1	3	20
*Enterobacter* spp.	8	11	2	4	0	8	2	0	3	0	19
*Pseudomonas* spp.	6	7	2	2	3	2	2	0	1	1	13
*Shigella* spp.	2	6	2	1	0	0	3	1	1	0	8
**Total**	**194**	**247**	**46**	**95**	**70**	**113**	**41**	**43**	**18**	**15**	**441**
	Df = 11, χ2 = 69.56, P < 0.0001		Df = 77, χ2 = 114.9, p-value = 0.003								

NLFGNR = Non-lactose ferment or gram negative rods.

When we compared the culture positivity of the samples, pus /discharge samples were 2.38 times positive for bacterial isolates than wound swab samples (OR = 2.38, 95%CI: 1.64-3.45, P < 0.0001). Females were found to be 5.16 times more at risk to get infection than males (OR = 5.16, 95% CI: 3.38-7.91, P < 0.0001). Age was also found to have significant association with a p-value of 0.003 [Table 4].

## Discussion

Among 628 study subjects, bacterial pathogens were isolated from 441-patients with the isolation rate of 70.2%. This was higher than the results reported by Mulu *et al.* in the same hospital [9]. This variation may be due to little variation in laboratory facilities that we have currently. Among the total isolates, 282 (63.9%) were gram positive and 159 (36.1%) were gram negative. The predominant isolates in the present study was found to be *S. aureus,* which was 32.9%, this was consistent with reports from India 30.1% and lower than Gondar hospital 65% [9, 10]. Although, *S. aureus* is predominant in both reports, the rate of isolation declined compared to the previous reports which was 65% [9]. This low rate of isolation may be due to improved facilities of the hospital management at present in the infection control program.

Most of the isolates were resistant to many of the antibiotics tested. The result showed that 66.2% of the isolates were resistant to tetracycline, followed 59.8% for ampicillin, 59.1% for cotrimoxazole, 51.7% for penicillin; least resistant being 6.3% for gentamycin. Among isolates tested, 18.3% of the isolates are becoming resistant to ceftriaxone and 33.3% for vancomycin. Among resistant isolates to more than one antibiotic, the most common pathogen was *Pseudomonas* spp., 100%; followed by S*. pyogenes* 85.8%; *Enterobacter* spp. 84.2%; and *E. coli* 83.4%. Fifty (11.3%) of the isolates were sensitive to all drugs tested and 68 (15.4%) were resistance to only one drug. However, among the total isolates, 323 (73.3%) were resistant for two or more drugs tested and 91 (20.6%) were resistant to more than 5 antibiotics tested. This finding was supported by other studies done in Ethiopia [11, 12].

The majority of the patients who were positive for wound culture were in the age range of 21 to 30 years, which was 113 (25.6%, followed by in the age range of 1-10 years, which was 95 (21.5%); age range of 11-20 which was 70 (15.9%). Among 441 isolates, 194/334 (58.1%) were from male and 247/284 (86.9%) were from female patients. These results demonstrate that being female is found a risk factor for getting an infection by bacteria. Females were found to be 5.16 times at risk to get infected by bacteria than males. When we compared the culture positivity of the samples, pus/discharge samples were 2.38 times positive for bacterial isolates than wound swab samples. As to the limitation of this study; since it is a retrospective study some of the data registered were not as to the standard and were not included. We also fail to include more variables because of unavailability.

## Conclusion

High prevalence of bacterial isolates were found from patients who provide pus and/or wound discharge sample in the study site *S. aureus* being the dominant. Most of the isolates were resistant to many of the antibiotics tested where all isolates of *Pseudomonas* spp. being resistant to two or more antibiotics. It is, therefore, necessary to do an antibiotic susceptibility test before drug prescription to effectively control wound infection.

## Abbreviations

CNS: Coagulase negative staphylococci
MDR: Multidrug resistant
WHO: World Health Organization.
